# Perimenopause Decreases SERCA2a Activity in the Hearts of a Mouse Model of Ovarian Failure

**DOI:** 10.3390/biom14060675

**Published:** 2024-06-09

**Authors:** Ciara Barry, Sarah Rouhana, Jessica L. Braun, Mia S. Geromella, Val A. Fajardo, W. Glen Pyle

**Affiliations:** 1 IMPART Team Canada Investigator Network, Dalhousie Medicine, Saint John, NB E2K 5E2, Canada; 2 Centre for Bone and Muscle Health, Brock University, St. Catharines, ON L2S 3A1, Canadavfajardo@brocku.ca (V.A.F.); 3 Department of Kinesiology, Brock University, St. Catharines, ON L2S 3A1, Canada; 4 Women’s Health Research Institute at BC Women’s Hospital + Health Centre, Vancouver, BC V6H 2N9, Canada

**Keywords:** perimenopause, calcium, menopause, phospholamban

## Abstract

Risk of cardiovascular disease mortality rises in women after menopause. While increased cardiovascular risk is largely attributed to postmenopausal declines in estrogens, the molecular changes in the heart that contribute to risk are poorly understood. Disruptions in intracellular calcium handling develop in ovariectomized mice and have been implicated in cardiac dysfunction. Using a mouse model of menopause in which ovarian failure occurs over 120 days, we sought to determine if perimenopause impacted calcium removal mechanisms in the heart and identify the molecular mechanisms. Mice were injected with 4-vinylcyclohexene diepoxide (VCD) to induce ovarian failure over 120 days, mimicking perimenopause. Hearts were removed at 60 and 120 days after VCD injections, representing the middle and end of perimenopause. SERCA2a function was significantly diminished at the end of perimenopause. Neither SERCA2a nor phospholamban expression changed at either time point, but phospholamban phosphorylation at S16 and T17 was dynamically altered. Intrinsic SERCA inhibitors sarcolipin and myoregulin increased >4-fold at day 60, as did the native activator DWORF. At the end of perimenopause, sarcolipin and myoregulin returned to baseline levels while DWORF was significantly reduced below controls. Sodium–calcium exchanger expression was significantly increased at the end of perimenopause. These results show that the foundation for increased cardiovascular disease mortality develops in the heart during perimenopause and that regulators of calcium handling exhibit significant fluctuations over time. Understanding the temporal development of cardiovascular risk associated with menopause and the underlying mechanisms is critical to developing interventions that mitigate the rise in cardiovascular mortality that arises after menopause.

## 1. Introduction

Early in life, women enjoy a relative cardiovascular advantage over men, with lower rates of cardiovascular disease morbidity and mortality [1]. However, with the onset of menopause, cardiovascular disease mortality rises more quickly in women than in age-matched men [1,2]. The postmenopausal rise in cardiovascular disease mortality has been ascribed to the decline in cardioprotective estrogens, although the specific biological mechanisms that increase mortality remain poorly understood [3].

For most women, menopause is not an abrupt event, but rather a physiological state following a perimenopausal transition lasting a decade or longer [4,5]. Furthermore, perimenopause is marked by fluctuations in circulating estrogens in a milieu of hormonal changes [4]. How these complex hormonal changes impact cardiac physiology has remained largely unexplored due to the lack of an animal model that temporally recapitulates the physiological alterations of perimenopause. The discovery that 4-vinylcyclohexene diepoxide (VCD) injections in micecan specifically target the ovaries and produce a perimenopausal state has opened the door for investigations of this important biological period [6]. We [7] and others [8,9,10] have shown that alterations in the cardiovascular system during perimenopause may lay the foundation for the postmenopausal increase in cardiovascular disease mortality seen in women.

Calcium acts as a trigger for cardiac muscle contraction. Tight regulation of intracellular calcium levels is required for normal heart function, and dysregulation can lead to dysfunction and disease by disrupting contractility and impacting intracellular signaling cascades. After menopause, women have a disproportionately increased risk of heart failure with preserved ejection fraction (HFpEF), a form of heart failure marked by impaired relaxation [11,12]. Women are also more likely to die following a heart attack than men [13,14], an outcome that is largely driven by sudden cardiac death [15,16,17]. While HFpEF and cardiac arrhythmias are distinct cardiac conditions, calcium dysregulation plays a key role in both. Specifically, alterations in calcium removal can induce intracellular calcium overload and contribute to the diastolic dysfunction of HFpEF, and cardiac arrhythmias resulting from ionic imbalance contribute to many post-acute myocardial infarction deaths. Studies using ovariectomized rodents show disruptions in intracellular calcium handling in the estrogen-deficient state meant to mimic menopause [18,19,20], but whether calcium removal changes occur during the perimenopausal transition has not been investigated.

Using the VCD model of ovarian failure, we examined changes in sarcoplasmic/endoplasmic reticulum Ca^2+^ATPase 2a (SERCA2a) [21] activity and calcium uptake in the hearts of mice as they transition through a perimenopausal phase. We show, for the first time, how calcium removal in the heart is negatively impacted during a perimenopausal phase, setting a foundation for worse cardiovascular disease outcomes after menopause, and providing a mechanistic explanation for the well-established rise in cardiovascular mortality that plagues postmenopausal women.

## 2. Methods

Animals. Sexually mature female CD-1 mice (78–105 days) were purchased from Charles River Laboratories (St. Constant, QC, Canada) and group-housed on a 12 h light/dark cycle. Food and water were provided ad libitum. Animals acclimatized to the animal facility for at least one week prior to use. All procedures were conducted in accordance with the guidelines set by the Animal Care and Use Committee of the University of Guelph and the Canadian Council on Animal Care.

VCD Mouse Model. A perimenopausal phase was induced using VCD injections as we have previously described [7]. Mice were injected daily (160 mg/kg, IP) with VCD (MilliporeSigma, Oakville, ON, Canada) for 15 consecutive days. VCD drives the loss of primary and primordial follicles in the ovary through atresia but leaves the rest of the ovary intact for production of non-estrogen hormones. Control mice were injected with vehicle (sesame oil).

Vaginal Cytology. Estrus cycling was monitored by vaginal cytology. Vaginal cells were examined under a light microscope to determine the estrous phase. Vaginal cytology was performed for 10 consecutive days between day 50 and 60 after the start of injections, and for at least 10 days prior to 120 days post-injections. Control females maintained regular estrous cycles of ~4–6 days in length. Mice that exhibited an irregular, prolonged estrous duration of >6 days in length at 60 days post-injections (VCD 60D) were used to represent the mid-point of perimenopause. When mice experienced 10 consecutive days of continuous diestrus, they were considered to be acyclic and in ovarian failure, described as being at the end of perimenopause. All mice were acyclical by day 120 (VCD 120D).

Myocardial Samples. Mice were euthanized using CO_2_. The hearts were quickly excised, rinsed in ice-cold saline, and stored at −80 °C.

SERCA2a Activity. Ionophore (A23187; Sigma Aldrich, St. Louis, MO, USA) supported and calcium-dependent SERCA2a ATPase activity was measured in cardiac muscle homogenates with an enzyme-linked spectrophotometric assay using an M2 Molecular Devices plate reader (Molecular Devices, San Jose, CA, USA) as previously described [22]. Briefly, cardiac muscle homogenates (150 g total protein), pyruvate kinase, and lactate dehydrogenase (18 U I^−1^ for both) were all added to SERCA activity buffer (20 mM HEPES, 200 mM KCl, 10 mM NaN_3_, 1 mM EGTA, 15 mM MgCl_2_, 5 mM ATP, 10 mM phosphoenolpyruvate). Subsequently, 4 L of 1.9% (w/v) NADH was added, and the plates were then read at 340 nm for 30 min. SERCA2a-dependent activity in cardiac muscle homogenates was calculated with a pathlength correction, extinction coefficient of NADH (6.22 mM), and the subtraction of background ATPase activity in the presence of 1 L of a SERCA-specific inhibitor, cyclopiazonic acid (40 mM) from total ATPase activity across a range of calcium concentrations (pCa 5.02–7.04). A bicinchoninic acid (BCA) assay was done to normalize SERCA2a activity to grams of protein. Maximal ATPase activity was obtained from the raw data, and the pCa_50_ or concentration required to elicit half V_max_ (in negative logarithm units) was obtained after fitting the data onto a sigmoidal dose–response curve using GraphPad Prism.

Calcium Uptake. ATP-dependent calcium uptake was measured in cardiac muscle homogenates with an Indo-1 calcium fluorophore-based assay as previously published [22,23]. Homogenates were added to a reaction buffer (200 mM KCl; 20 mM HEPES; 10 mM NaN_3_; 5 M N,N,N′,N′-tetrakis(2-pyridylmethyl) ethylenediamine; 15 mM MgCl_2_, pH 7.0) along with Indo-1 (4 M final concentration; 50041, Biotum, Fremont, CA, USA). Calcium uptake was initiated with the addition of 4 L of ATP (250 mM, pH 7.0). A Molecular Devices M2 plate reader was used to measure fluorescence of calcium-bound Indo-1 (405 nm emission) and calcium-free Indo-1 (485 nm emission) upon excitation at 355 nm at 37 °C. The ratio of calcium-bound to calcium-free Indo-1 (405/485 nm) was used as an indicator of free calcium across time (0–1000 s). The rates of calcium uptake were obtained during the linear phase of uptake (150–400 s) and were normalized to mg of protein measured using a BCA assay. 

Immunoblotting. Samples of ventricular tissue were homogenized in ice-cold Standard Buffer (60 mM KCl; 30 mM Imidazole (pH 7.0); 2 mM MgCl_2_) containing protease (0.1 mM phenylmethylsulfonyl fluoride; 0.01 mM leupeptin; 0.2 mM benzamidine) and phosphatase inhibitors (P0044, MilliporeSigma, Oakville, ON, Canada). Protein concentration was determined using a Bradford assay (Bio-Rad Laboratories Ltd., Mississauga, ON, Canada). Samples were separated using SDS-PAGE with resolving gels of various percentages (5–16%) and proteins transferred to nitrocellulose membrane. Phospholamban, sarcolipin, myoregulin, and Dwarf Open Reading Frame (DWORF) immunoblots were done following a 50 min room temperature transfer at 300 mA; SERCA2a and sodium-calcium (Na^+^–Ca^2+^) exchanger blots were done using a 100 V transfer for 2.5 h at room temperature, as we have done previously [7,24]. Membranes were blocked for 1 h at room temperature in Tris-buffered saline (0.01 M Tris-base; 0.15 M NaCl, pH 7.6) with 5% dry milk powder or 5% bovine serum albumin (phospholamban blots only). Membranes were probed with primary antibodies at various dilutions for ~18 h at 4 °C (Table 1). Horseradish peroxidase-conjugated secondary antibodies (1:5000 for all except, 1:10,000 for phosphorylated phospholamban T17; MilliporeSigma, Oakville, ON, Canada) were used for 1 h at room temperature. Protein bands were detected using Signal Fire Elite ECL Reagent (Cell Signaling Technology, #12757, Danvers, MA, USA) and imaged using a Bio-Rad Imaging MP system (Bio-Rad Laboratories Ltd., Mississauga, ON, Canada). The bands were analyzed using ImageJ software (ImageJ 1.53a, National Institutes of Health, Bethesda, MD, USA). Ponceau staining was used to determine protein load across lanes, and immunoblot values were normalized to total protein load.

Statistical analysis. All data are shown as the mean ± standard error of mean (SEM). Statistical analyses were done using one-way ANOVA followed by a post-hoc Fisher’s Least Significant Difference. *p* < 0.05 was considered statistically significant.

## 3. Results

**Figure 1:**
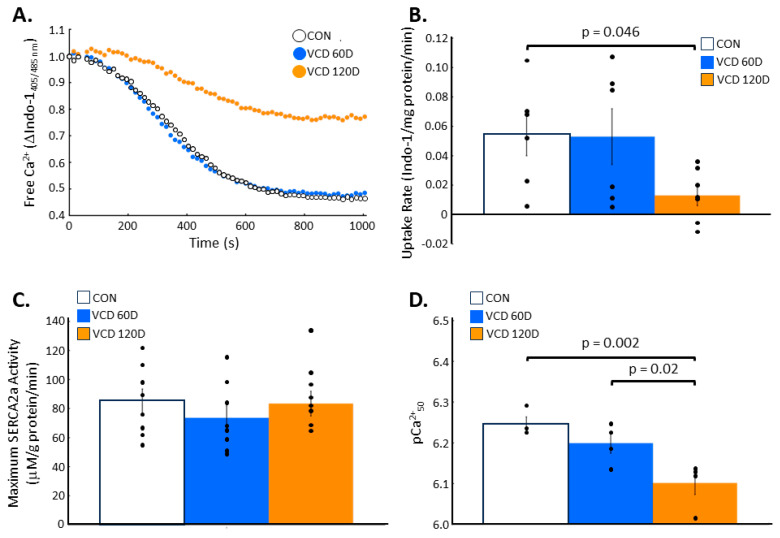
SERCA2a function is decreased during the perimenopausal transition. (**A**) SERCA2a uptake of calcium over time was not altered in VCD 60D ventricular homogenates, while VCD 120D samples had a significant slowing of SERCA2a activity. (**B**) Mean calcium uptake rate by SERCA2a was not significantly different in VCD 60D samples compared to that in controls but was significantly decreased by day 120. (**C**) SERCA2a activity in the presence of maximally activating calcium was not different in either VCD group compared to that in control. (**D**) Calcium sensitivity of SERCA2a exhibited an insignificant decline in VCD 60D samples and a further significant decrease in VCD 120D homogenates. **Key:** CON, control; VCD 60D, samples taken at 60 days after the start of VCD injections; VCD 120D, samples taken 120 days after start of VCD injections. All data are presented as the mean ± SEM. N = 4 to 8 in each group.

Calcium uptake and SERCA2a affinity for calcium declines by the end of perimenopause. SERCA2a-mediated calcium uptake in cardiac homogenates taken from control or VCD-injected mice was monitored over time (Figure 1A). While calcium uptake in samples from VCD 60D hearts exhibited a similar time course as those from control mouse hearts, cardiac homogenates taken at 120D post-VCD injection had a drastically reduced calcium uptake profile. In turn, the rate of calcium uptake obtained during the linear portion of the curve (150–400 s) in control and VCD 60D hearts were 0.055 ± 0.015 and 0.053 ± 0.019 per mg protein per min, respectively (Figure 1B). VCD 120D hearts exhibited a ~76% decline in calcium uptake rate compared to that in controls, with a mean rate of 0.013 ± 0.007 per mg protein per min. When assessing SERCA2a activity, or the ability of SERCA2a to hydrolyze ATP, there were no differences found in maximal SERCA2a activity (Figure 1C). However, *p*Ca_50_—a measurement of calcium sensitivity—decreased from 6.25 ± 0.02 in control samples to 6.20 ± 0.02 at VCD 60D, indicative of a greater calcium requirement to elicit half V_max_, albeit this difference was not statistically significant (Figure 1D). In contrast, in the 120D VCD group, the decline in calcium sensitivity reached 6.10 ± 0.03, which was significantly different from both control (*p* = 0.002) and VCD 60D (*p* = 0.02) hearts. Together these data show that the physiological performance of SERCA2a was significantly impaired by the end of the perimenopausal phase, with a reduction in calcium uptake presumably due to a decrease in calcium sensitivity.

**Figure 2:**
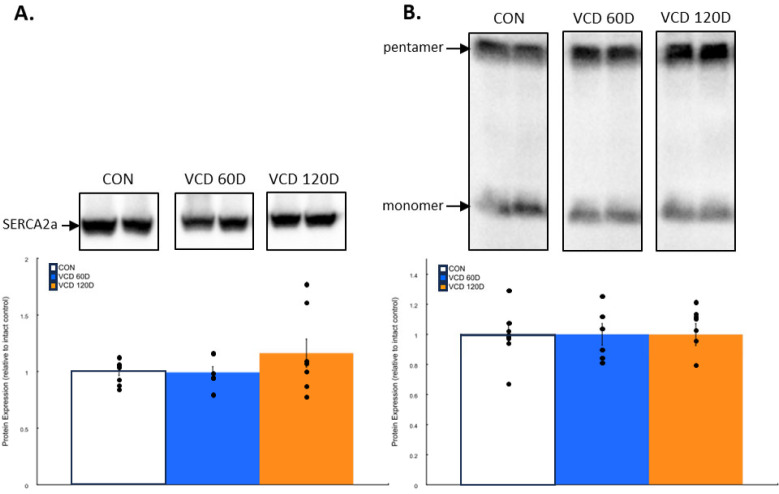
Perimenopause does not impact SERCA2a or phospholamban expression in the heart. (**A**) SERCA2a or (**B**) phospholamban expression. There were no significant differences in the expression of SERCA2a or phospholamban in VCD 60D or VCD 120D compared to those in controls. **Key:** CON, control; VCD 60D, samples taken at 60 days after the start of VCD injections; VCD 120D, samples taken 120 days after start of VCD injections. All data are presented as the mean ± SEM. N = 6 to 8 in each group.

SERCA2a and phospholamban expression are unaffected by perimenopause. A decline in SERCA2a function could be explained by reduced levels of SERCA2a, the dominant SERCA isoform in cardiac muscle [21], or increased expression of its regulatory peptide phospholamban. Immunoblotting of cardiac homogenates revealed no significant differences in SERCA2a expression at either perimenopausal time point compared to that in control samples (Figure 2A). Similarly, phospholamban expression remained consistent throughout the perimenopausal transition (Figure 2B).

**Figure 3:**
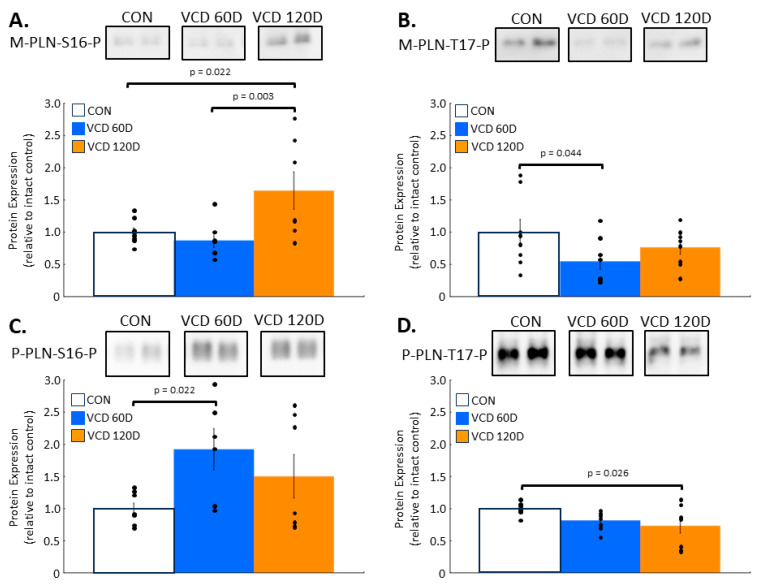
Phospholamban phosphorylation is affected by the ovarian failure of perimenopause. (**A**) Monomeric forms of phospholamban had increased phosphorylation of S16 at VCD 120D compared to that in controls. (**B**) At VCD 60D, T17 phosphorylation decreased in monomeric phospholamban relative to control levels. (**C**) Pentameric phospholamban had a significant increase in the phosphorylation of S16 at VCD 60D, which tended to decline towards control levels by VCD 120D. (**D**) T17 phosphorylation in pentameric phospholamban declined below baseline levels by VCD 120D. **Key:** CON, control; VCD 60D, samples taken at 60 days after the start of VCD injections; VCD 120D, samples taken 120 days after start of VCD injections. M-PLN, monomeric phospholamban; P-PLN, pentameric phospholamban. All data are presented as the mean ± SEM. N = 6 to 8 in each group.

Phospholamban phosphorylation is dynamically altered by perimenopause. Phospholamban regulation of SERCA2a activity is affected by phosphorylation at two known sites, both of which decrease inhibition and allow for an increase in SERCA2a activity and calcium uptake. Monomeric phospholamban exhibited a 64.5 ± 29.1% increase in S16 phosphorylation in 120D VCD samples compared to that in controls (*p* = 0.022) and was on average 77% higher than in 60D VCD samples (*p* = 0.003) (Figure 3A). T17 phosphorylation of monomeric phospholamban declined to 54.9 ± 12.5% of control levels by 60D post-VCD injections (*p* = 0.044) and remained 23.6 ± 10.6% below control levels by 120D VCD, which was not significantly different (Figure 3B).

In contrast to monomeric phospholamban, S16 phosphorylation increased 92.4 ± 32.0% above controls in 60D VCD hearts (*p* = 0.022), but by 120D, this change was 50.5 ± 33.9% above control levels, which was not significantly different (Figure 3C). T17 phosphorylation also exhibited a different temporal pattern as the 14.8 ± 5.1% decrease in phosphorylation seen in 60D VCD samples was not significant, but by 120D VCD, phosphorylation had declined by 23.6 ± 10.6% below control levels (*p* = 0.026) (Figure 3D).

These data demonstrate significant changes in phospholamban phosphorylation throughout the perimenopausal transition, which has not previously been investigated.

**Figure 4:**
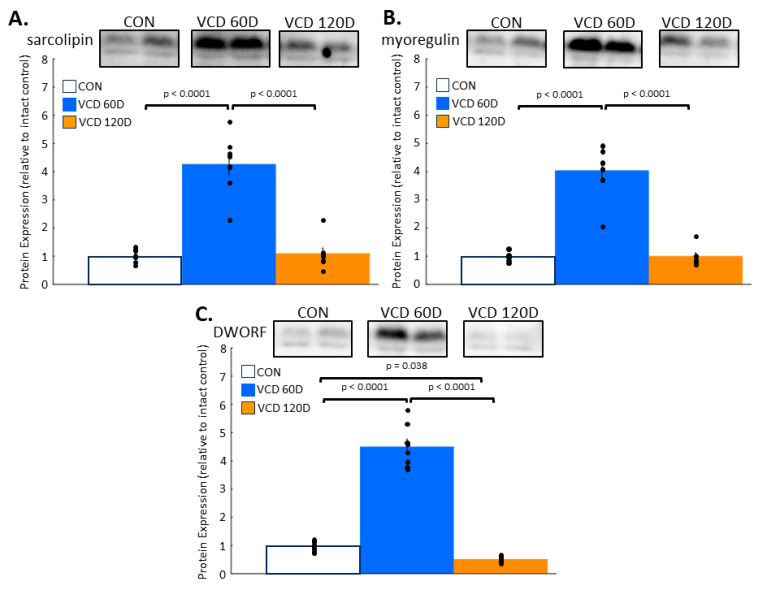
Peptide regulators of SERCA2a activity are differentially expressed across perimenopause. (**A**) Sarcolipin increased above control levels in VCD 60D samples and returned to baseline levels by VCD 120D. (**B**) Myoregulin exhibited a similar transient increase at VCD 60D that normalized by VCD 120D. (**C**) DWORF levels rose significantly above control levels by VCD 60D and fell below controls at VCD 120D. **Key:** CON, control; VCD 60D, samples taken at 60 days after the start of VCD injections; VCD 120D, samples taken 120 days after start of VCD injections. DWORF, Dwarf open reading frame. All data are presented as the mean ± SEM. N = 8 in each group.

Peptide regulators of SERCA2a activity exhibit dynamically altered expression in perimenopause. In addition to phospholamban, there are several other peptide regulators of SERCA. Sarcolipin inhibits SERCA activity directly and indirectly through phospholamban [25], while myoregulin has similar inhibitory effects through direct SERCA binding [26]. By contrast, DWORF enhances SERCA function by displacing the inhibitor regulators phospholamban, sarcolipin, and myoregulin [27]. Immunoblotting revealed significant increases over control levels that exceeded 4-fold changes in the expression of sarcolipin (Figure 4A), myoregulin (Figure 4B), and DWORF (Figure 4C) by 60D VCD (*p* < 0.0001 for all). By the end of the perimenopausal phase, the expression of sarcolipin and myoregulin had returned to levels similar to those of controls, while DWORF was 49.5 ± 3.2% lower than control levels (*p* = 0.038). Given DWORF’s stimulatory effect on SERCA activity, the reduced expression seen at 120D VCD was consistent with the reduced calcium removal activity we found at the same timepoint.

**Figure 5:**
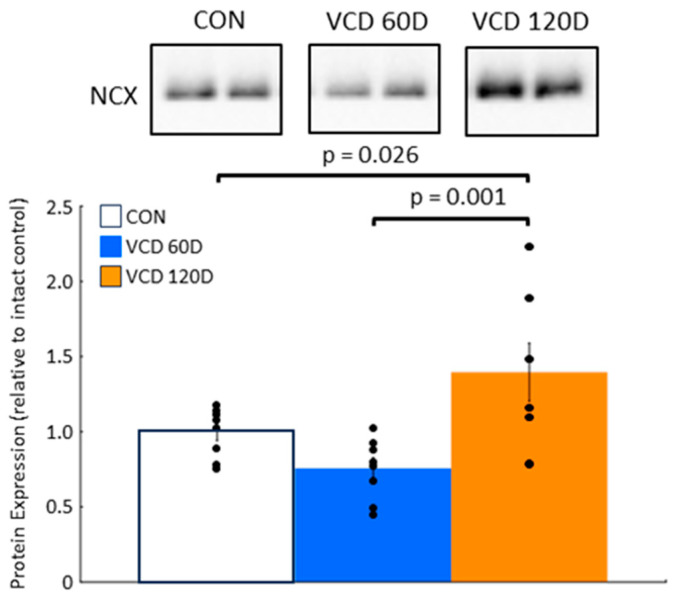
Na^+^–Ca^2+^ exchanger expression increases at the end of perimenopause. Na^+^–Ca^2+^ exchanger expression in VCD 60D samples was unaffected. By VCD 120D, the expression had risen to levels higher than that in controls. **Key:** CON, control; VCD 60D, samples taken at 60 days after the start of VCD injections; VCD 120D, samples taken 120 days after start of VCD injections. NCX, Na^+^–Ca^2+^ exchanger. All data are presented as the mean ± SEM. N = 8 in each group.

Na^+^–Ca^2+^ exchanger expression is increased at the end of perimenopause. Reduced SERCA2a function exposes cardiomyocytes to the risk of calcium overload. In stressed hearts, increased expression of the sarcolemmal Na^+^–Ca^2+^ exchange is a mechanism that can acutely compensate for impaired sarcoplasmic reticulum calcium removal. In 60D VCD samples, Na^+^–Ca^2+^ exchanger expression declined by 24.4 ± 7.1%, although this change did not reach statistical significance (*p* = 0.14) (Figure 5). In 120D VCD samples where SERCA2a-mediated calcium uptake was noted to be significantly reduced, Na^+^–Ca^2+^ exchanger expression increased by 39.6 ± 19.3% over control levels (*p* = 0.026). These data suggest that the calcium overload produced by reduced SERCA2a activity was compensated for by increasing Na^+^–Ca^2+^ exchanger expression to eject calcium into the extracellular environment.

## 4. Discussion

**Figure 6:**
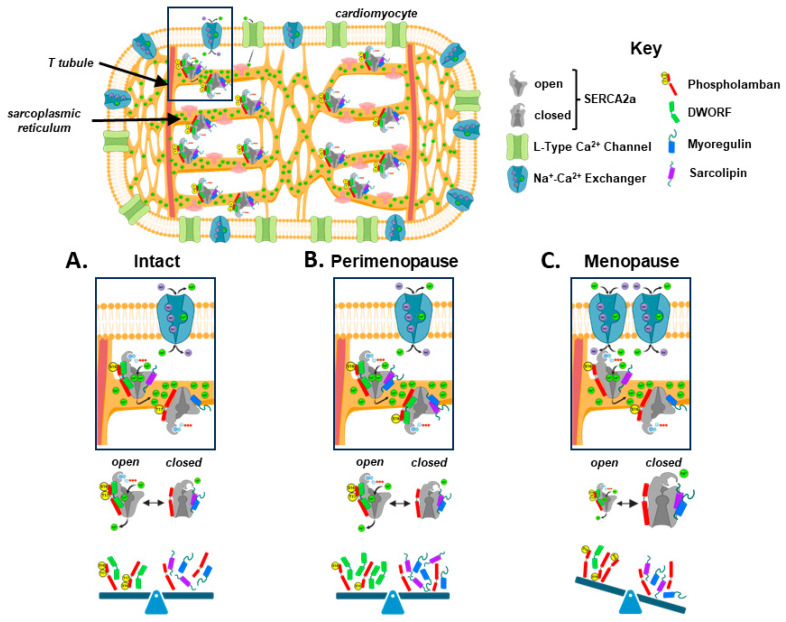
A model of SERCA2a regulation and intracellular calcium removal in a mouse model of perimenopause. (**A**) Murine myocardium maintains SERCA2a activity through a balanced regulation by phospholamban, sarcolipin, myoregulin, and DWORF, and secondary calcium removal by the Na^+^–Ca^2+^ exchanger. (**B**) At the mid-point of the perimenopausal transition, SERCA2a function is maintained with stimulation by increased pentameric phospholamban phosphorylation at S16, myoregulin expression, and sarcolipin levels, countered with inhibition of SERCA activity by reduced T17 phosphorylation of monomeric phospholamban and increased DWORF levels. (**C**) By the end of perimenopause, SERCA2a activity is significantly reduced, necessitating an increased reliance on removal by Na^+^–Ca^2+^ exchangers. Monomeric phospholamban phosphorylation at S16 stimulates SERCA2a function, but this drive is more than countered with a decline in pentameric phospholamban phosphorylation at T17 and a significant reduction in DWORF expression. Figure created with Biorender.

Understanding the cardiac risks that increase in women after menopause has been significantly limited by the lack of an animal model that recapitulates the physiological changes of menopause in a chronologically relevant manner. In the current study, we used a mouse model of gradual ovarian failure to show for the first time that SERCA2a function in the heart is significantly affected, even before the postmenopausal period (Figure 6). Specifically, a significant reduction in SERCA2a-mediated calcium uptake was found, presumably due to a reduction in its affinity for calcium. These changes to SERCA2a function were accompanied by profound changes in the expression of DWORF, myoregulin, and sarcolipin, and the dynamic alteration of phospholamban phosphorylation. Concomitant with decreased SERCA2a function, we detected an increase in Na^+^–Ca^2+^ exchanger expression, which may represent a compensatory response to calcium stress that ultimately proves unsuccessful in the chronic adaptation to stress [28]. Together these data represent the first investigation of intracellular calcium handling changes that evolve during the perimenopausal transition and present a mechanism for the increase in cardiovascular disease mortality that is well characterized in postmenopausal women. Understanding the molecular changes that occur in the heart in association with menopause is critical to identifying women most at risk for cardiovascular disease and to creating therapeutic interventions that have a rational basis for use.

Calcium-Dependent Mechanisms of Risk. Menopause does not cause cardiovascular disease, but it is a known risk factor. After menopause, women are more likely to develop HFpEF than men of a similar age [29,30], and their risk of post-acute myocardial infarction mortality is also significantly higher than that of age-matched men [31]. While these outcomes are distinct and likely driven by multiple factors, the decrease in SERCA2a function could contribute to an elevated risk of both. The reduction in calcium removal by SERCA2a leads to a prolonged and impaired relaxation phase that can ultimately manifest as diastolic dysfunction [32]. Furthermore, elevated intracellular calcium stimulates calcium-dependent signaling molecules that are critical in the myocardial hypertrophy process that characterizes HFpEF [30]. The increased reliance on Na^+^–Ca^2+^ exchangers to remove intracellular calcium is an imbalanced mechanism that results in the net gain of positive charges in the cell. This imbalance is known to increase the risk of cardiac arrhythmias [33], a common cause of acute post-myocardial infarction mortality [15,16,17]. While the changes in calcium handling could drive an increased risk for these pathologies, it should be noted that menopause itself is generally not sufficient to cause cardiac disease. Instead, the manifestation of heart failure or cardiac arrhythmias typically requires additional stressors such as diabetes, hypertension, or obesity, all of which have increased rates of occurrence after menopause. The underlying alterations in calcium handling simply act as a foundational change upon which cardiovascular disease risk is built. A significant finding of this study is the developmental timing of these risk factors, evolving during the perimenopausal period. This relatively early change in the perimenopausal transition suggests that strategies to mitigate cardiovascular disease risk may need to be considered even before the onset of menopause.

SERCA2a Regulation. SERCA activity is regulated through several mechanisms including differential protein expression; post-translational modifications including oxidation, acetylation, glutathioylation, SUMOylation, and phosphorylation; and peptide or protein binding [34,35]. Our study found no change in the expression of SERCA2a or phospholamban at any point, eliminating the loss of SERCA2a or increased expression of phospholamban as explanations for the observed changes in SERCA2a function. How other post-translational modifications may impact SERCA2a and its calcium removal activity remains to be investigated.

The phosphorylation of phospholamban by protein kinase A (PKA) at S16 relieves the inhibition of SERCA2a to allow for increased activity, as does the phosphorylation of T17 by calcium-calmodulin-dependent protein kinase, although T17 phosphorylation may be less potent [36]. The regulatory effects of phospholamban have historically been ascribed to the monomeric form, but recent work by Funk and colleagues [37] and others [38] suggest that pentameric phospholamban may also impact SERCA2a function in vivo. They found that pentameric phospholamban is an effective competitor for PKA and that the phosphorylation by PKA could enhance cardiac contractility in a manner similar to that of phospholamban knockout mice [37]. Our data show that monomeric phospholamban phosphorylation at S16 increased at the end of the perimenopausal phase, while T17 phosphorylation transiently declined in the middle of this transitional phase. These modifications predict an increase and decrease in SERCA2a activity, respectively, neither of which manifested at that time point. Pentameric phospholamban exhibited an almost mirror image, consistent with the competitive model proposed by Funk et al. [37]: S16 phosphorylation increased in the middle of the perimenopausal phase and T17 phosphorylation decreased by the end. The increase in S16 phosphorylation is predicted to increase SERCA activity at VCD 60D, but no detectable change in the calcium removal rate suggests that this affect is antagonized, possibly by the simultaneous decline in monomeric T17 phosphorylation. Our study also presents the first report of a change in T17 phosphorylation in pentameric phospholamban, specifically a significant decline at the end of perimenopause. While the functional effects of pentameric phospholamban phosphorylation at T17 are not known, applying the model proposed by Funk and colleagues [37] allows for a predicted decrease in SERCA2a activity. It should be noted that the concomitant increase in S16 phosphorylation in the phospholamban monomers could offset this effect given the larger and more potent change.

We also examined changes in other peptide regulators of SERCA to explain the decline in calcium removal at the end of perimenopause. The peptides sarcolipin [39] and myoregulin [40] bind to SERCA and decrease its activity, while DWORF has an opposing effect by promoting sarcoplasmic reticulum calcium re-uptake [27]. Like the phospholamban phosphorylation patterning, there were changes at VCD 60D with opposing effects: the SERCA inhibitors myoregulin and sarcolipin increased, as did the activator DWORF. The lack of any functional change again suggests a relative balancing of stimulatory and inhibitory pathways across all regulators (including phospholamban). By contrast, at day 120 post-VCD injections, both sarcolipin and myoregulin had returned to baseline levels, while DWORF exhibited a significant decline. The net loss of a SERCA2a activator with decreased DWORF is consistent with the reduction in SERCA2a function seen at the end of perimenopause. This change provides a mechanistic basis for our observed physiological change, although it is not clear if this is the only driver of diminished calcium uptake and SERCA2a affinity for calcium at this point of the transition.

An interesting and novel finding of our study is the discovery of myoregulin expression in murine ventricles. Myoregulin is more widely recognized as a skeletal muscle peptide, with previous studies reporting no detectable cardiac expression [40]. More recently, Appleby and colleagues [41] reported myoregulin mRNA in mouse hearts, but our study is the first to show protein expression of this SERCA regulator in ventricular samples. Whether these results are specific to mouse hearts or represent a new discovery that is the result of an antibody capable of detecting myoregulin across species is not clear, and additional studies should be undertaken to investigate the expression of myoregulin in the hearts of different species.

Na^+^–Ca^2+^ Exchanger. The Na^+^–Ca^2+^ exchanger is a bidirectional ionic regulator whose primary physiological function is to contribute to calcium entry (reverse mode) during the depolarizing phase of a myocyte action potential, followed by calcium extrusion (forward mode) late to facilitate relaxation. Under physiological conditions, the exchanger is a secondary mechanism for calcium removal, with SERCA2a acting as the primary removal tool [42]. Under pathological stress, Na^+^–Ca^2+^ exchanger expression is acutely increased to produce a two-fold effect: aid in calcium removal through its forward direction as SERCA2a is overwhelmed [43] and paradoxically facilitate calcium loading of the sarcoplasmic reticulum [44]. These changes in Na^+^–Ca^2+^ exchanger activity appear to be ultimately unfavorable, and a chronic increase in its expression contributes to pathology, specifically HFpEF. Chronically increased expression or activity can couple with sodium overload to drive the exchanger in the forward direction, exacerbating calcium overload [45] and also reducing the release of calcium from the sarcoplasmic reticulum by diminishing the amount of trigger calcium that remains in the cytosol after entry through L-type calcium channels [46]. Studies show that inhibition of the exchanger’s activity mitigates the development of HFpEF [45].

In our study, the upregulation of the Na^+^–Ca^2+^ exchanger during perimenopause could represent an attempt to compensate for the calcium overload resultant from reduced SERCA2a function. While this response initially tempers calcium overload, it could ultimately set the stage for an increased risk of HFpEF development, which is elevated after menopause. Furthermore, the non-neutral exchange of ions driven by the disturbance of these pumps and exchangers could put cardiac myocytes closer to the threshold for cardiac arrhythmias, which is a known risk for mortality acutely following myocardial infarctions.

## 5. Conclusions

The elevated threat of cardiovascular disease morbidity and mortality faced by women after menopause has long been recognized, but its mechanistic basis remains poorly understood. The development of an animal model that recapitulates many of the key changes that occur with menopause in a temporally appropriate manner represents a significant opportunity to close the knowledge gap in women’s heart health. Understanding the mechanisms by which cardiovascular disease risk rises in women after menopause is critical to identifying women who are most at danger of disease and death and to the development of therapies that mitigate risk by precisely targeting those changes that drive disease.

Our current study is the first to show that intracellular calcium removal by SERCA2a, a critical player in cardiac physiology and pathophysiology, is significantly diminished by the end of an ovarian failure stage that mimics perimenopause. This disruption in calcium handling creates a disequilibrium that is consistent with increased risks of HFpEF and cardiac arrhythmias, two more common conditions in women after menopause. Our work identifies possible mechanisms for increased cardiovascular mortality in women after menopause and offers potential therapeutic targets to mitigate risk in postmenopausal women. Moreover, the disruptions in calcium handling in the heart during perimenopause suggests that cardiovascular disease risk develops and may even be established before the onset of menopause. This unexpectedly early response to the changes in ovarian estrogens provides support for the idea of perimenopausal interventions to reduce or slow the establishment of risk rather than a potentially futile strategy to reverse changes with postmenopausal hormone supplementation.

## Figures and Tables

**Table 1:** Primary antibodies used for immunoblotting.

Antigen	Species and Product Number	Company	Dilution
SERCA2a	anti-rabbit, 4388S	Cell Signaling	1:1000
PLN	anti-rabbit, 9608305	MyBioSource	1:5000
P-PLN-T17	anti-rabbit, 4756839	MyBioSource	1:5000
P-PLN-S16	anti-rabbit, 9143798	MyBioSource	1:5000
NCX	anti-rabbit, 79350S	Cell Signaling	1:1000
Myoregulin	anti-rabbit, 5400549	MyBioSource	1:1000
Sarcolipin	anti-rabbit, 713457	MyBioSource	1:1000
DWORF	anti-rabbit, 541442	MyBioSource	1:1000

Key: PLN, phospholamban; NCX, sodium-calcium exchanger.

## Data Availability

The original contributions presented in the study are included in the article/Supplementary Material, further inquiries can be directed to the corresponding author/s.

## Supplementary Materials

The following supporting information can be downloaded at: https://www.mdpi.com/article/10.3390/biom14060675/s1.
