# Supplementary figures and images for: Perimenopause Decreases SERCA2a Activity in the Hearts of a Mouse Model of Ovarian Failure
Source: Biomolecules. 2024 Jun 9;14(6):675. doi: 10.3390/biom14060675 (PMC11201532; doi:10.3390/biom14060675)

Fig.2A

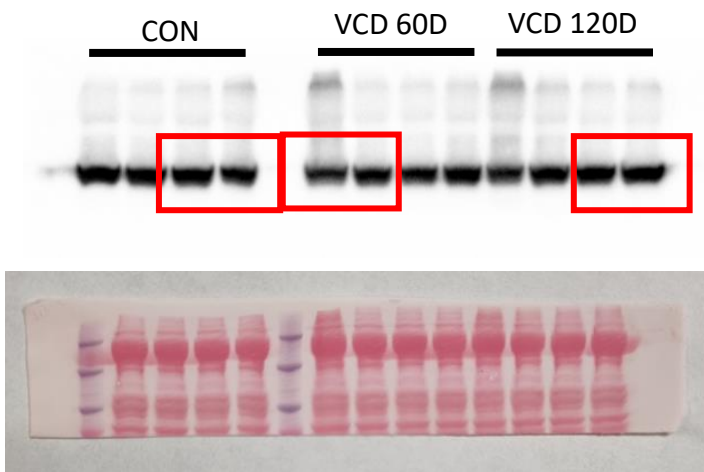

Fig.2B

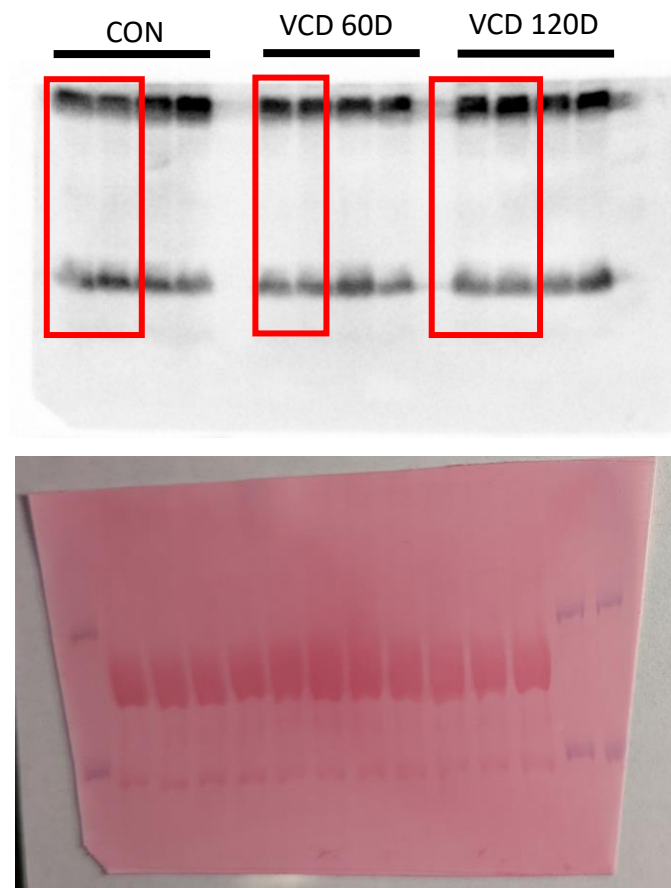

Fig.3A

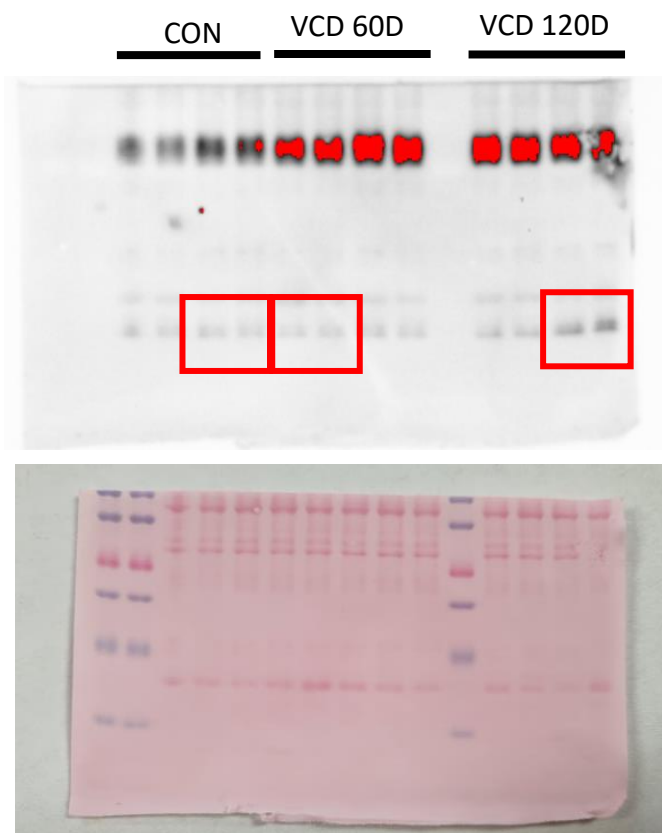

Fig.3B

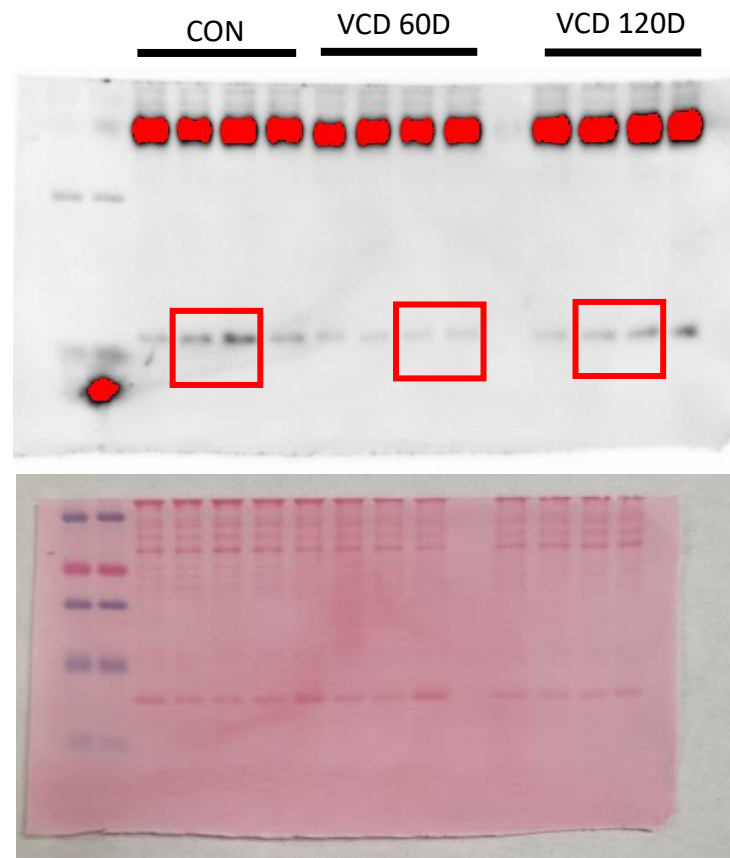

CON      VCD 60D      VCD 120D

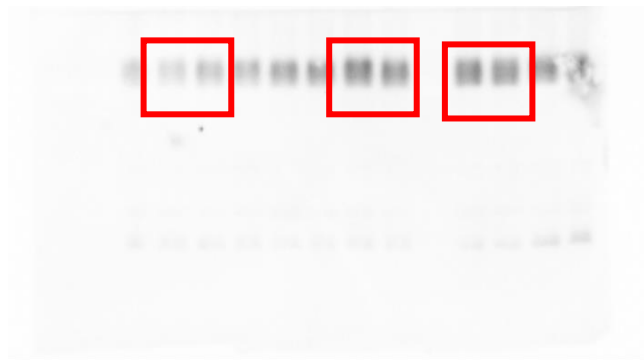

Fig.3C

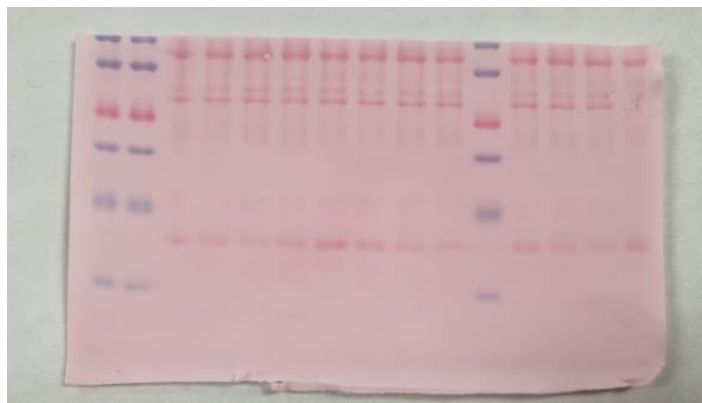

CON      VCD 60D      VCD 120D

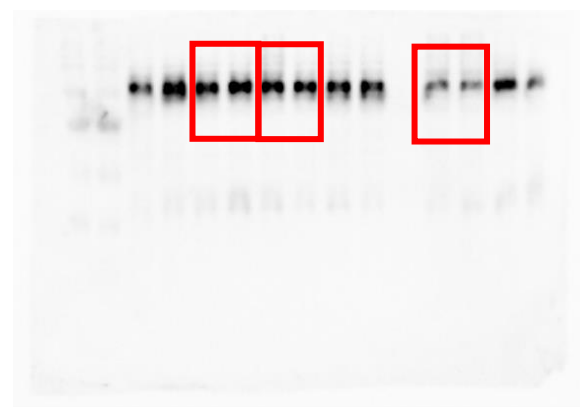

Fig.3D

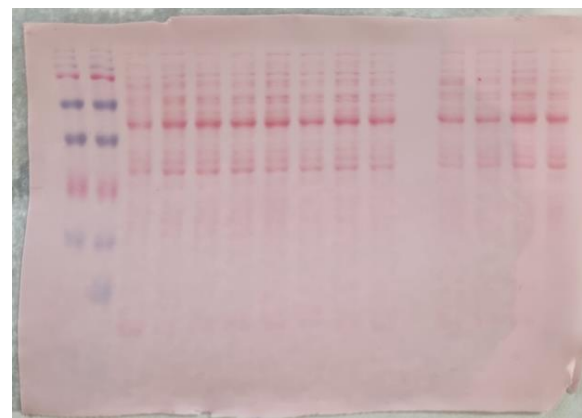

Fig.4A

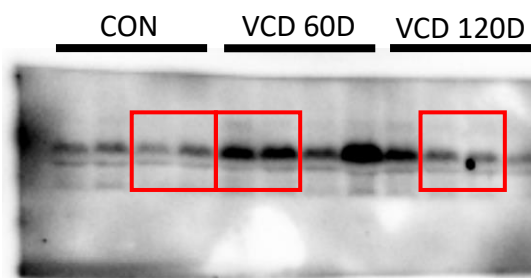

Fig.4B

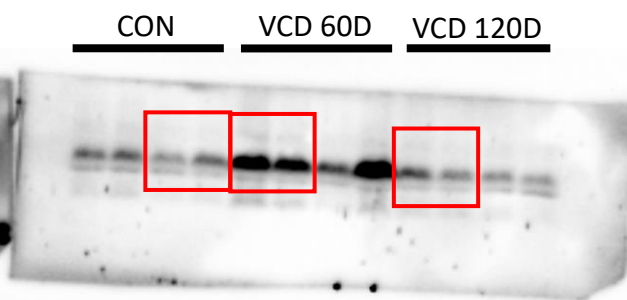

Fig.4C

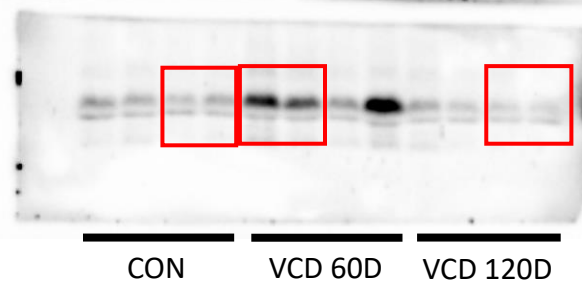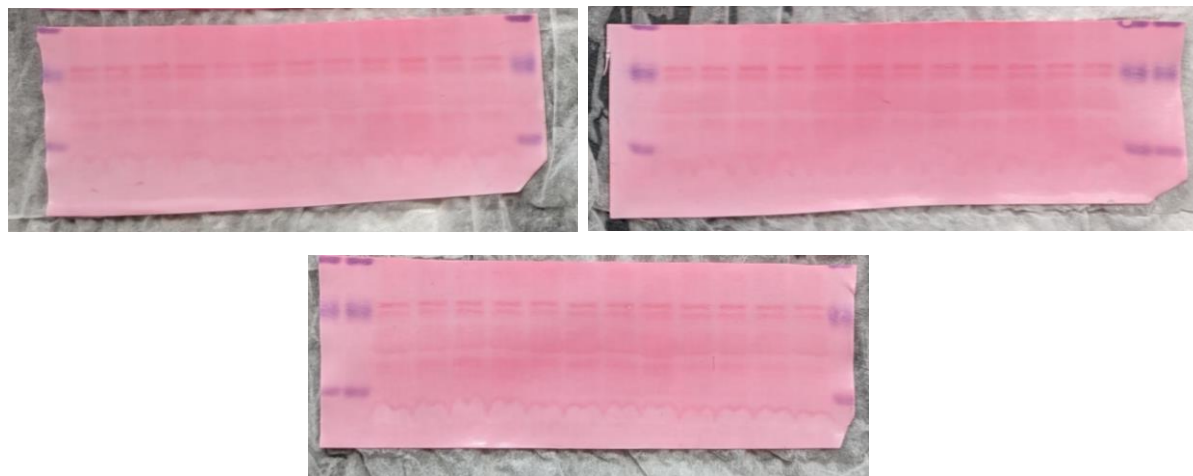

Fig.5

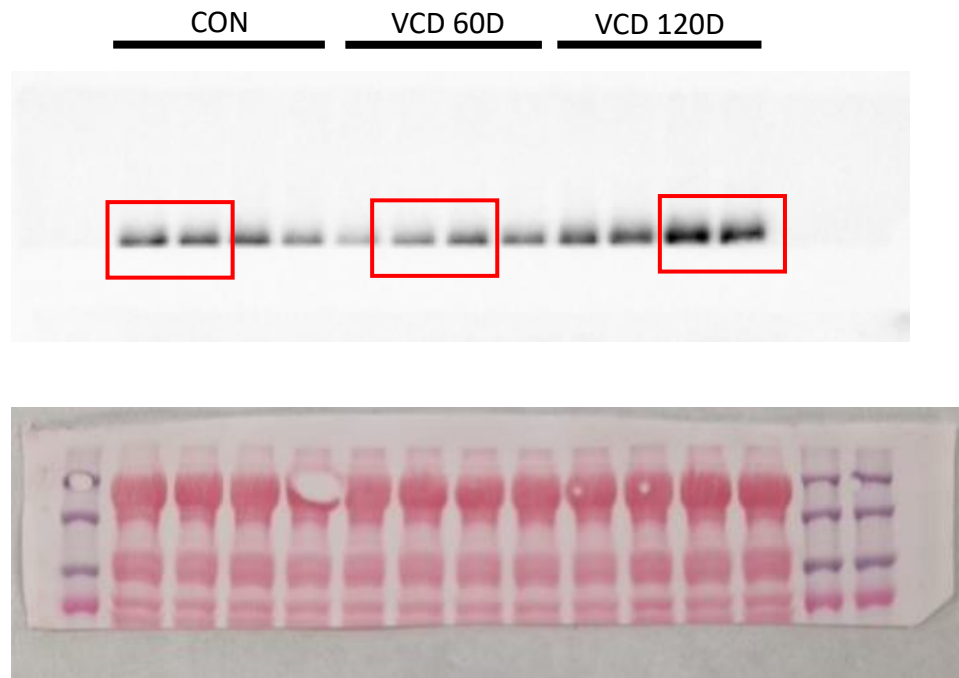

Supplement: Supplementary file 1 [file biomolecules-14-00675-s001.zip › biomolecules-3030869-supplementary.pdf]
